# Nutritional Composition and Antinutritional Factors of Five Wild Edible Fruits Grown in the Mekdela District, South of Wollo, Ethiopia

**DOI:** 10.1155/2024/9980936

**Published:** 2024-03-11

**Authors:** Yalew Yiblet

**Affiliations:** Department of Biology, College of Natural and Computational Sciences, Mekdela Amba University, P.O. Box 32, Tuluawlia, Ethiopia

## Abstract

This study was carried out to determine the mineral content and nutritional properties of five wild fruits *Rhus vulgaris*, *Rosa abyssinica*, *Rhus natalensis*, *Euclea racemosa*, and *Ficus sur*. The proximate composition parameters (moisture, ash, crude fiber, crude fat, and crude protein) and antinutritional factors were evaluated using methods of the Association of Official Analytical Chemists and elemental analysis using the atomic absorption spectroscopy technique. Among the five wild edible fruit species, *Rhus vulgaris* had the highest carbohydrate content (83.3 ± 0.28 g/100 g) and a high total energy (344.5 ± 2.21 kcal/100 g). *Euclea racemosa* was found to have the maximum ash content (12.8 ± 0.37 g/100 g), protein content (3.22 ± 0.01 g/100 g), and moisture (16.24 ± 0.003 g/100 g), respectively. *Rhus natalensis* showed the highest fiber content (9.54 ± 0.003 g/100 g). Mineral analysis showed that local wild fruits contained a considerable amount of minerals. The calcium concentration ranged from 99.51 mg/100 g in *Euclea racemosa* to 160.12 mg/100 g in *Ficus sur*. Potassium concentration varied from 54.34 mg/100 g in *Euclea racemosa* to 234 mg/100 g in *Rhus vulgaris*. Iron ranges from 21.4 mg/100 g in *Rosa abyssinica to* 41 mg/100 g in *Rhus natalensis*, and zinc ranges from 2.3 mg/100 g in *Rhus vulgaris* to 4.2 mg/100 g in *Ficus sur.* A high saponin content (2.12 mg/100 g) and a low tannin content (0.23 mg/100 g) were obtained in *Rosa abyssinica.* The phytate content (1.52 mg/100 g) and the oxalate content (0.9 mg/100 g) were high in *Rhus natalensis.* In conclusion, the present study shows that wild fruits can be used as food supplementation in food in a safe area.

## 1. Introduction

Many kinds of edible wild fruits are eaten as a source of nutrition in poor nations. Some people regularly gather edible fruits and other plants from natural environments to meet their appropriate nutritional needs, due to factors such as rapid population growth, lack of arable land, and high prices of readily available staples [1]. In addition to providing humans with vital nutrients such as carbohydrates, dietary fiber, and protein, these wild fruits also include minerals and phytochemicals that are good for your health [2]. Due to their high fiber and antioxidant content, recent studies have revealed that wild fruits can treat a variety of illnesses, including diabetes, cardiovascular problems, inflammations, and disorders of the digestive and urinary tract [3].

Antioxidants is another essential element that reduces the risk of developing chronic diseases by eliminating free radicals that have formed in different tissues and shielding those tissues from oxidative damage [2]. To improve dietary diversity, the Food and Agriculture Organization (FAO) of the United Nations is actively advocating for the conservation and sustainable use of biodiversity for agriculture and nutrition [4]. However, the main problem that prevents some of these wild fruits from being fully used is the presence of certain antinutrients, which can decrease mineral bioavailability and digestibility, particularly protein and carbohydrates [5]. Ethiopia, along with many other African nations, is extremely fortunate to have an abundance of fruit-bearing species that produce domesticated and wild fruits throughout the year [6]. Unfortunately, nutritional deficiencies remain a widespread and worrying problem in the nation, particularly affecting young people and expectant mothers [7].

Much attention has recently been paid to the evaluation of different wild edible fruits because they are an essential part of the human diet, providing the body with energy, protein, and essential vitamins and minerals [8]. It was noted in most of these correspondences that the nutritional value of foods made from unusual plants may occasionally be better than that of ordinary vegetables. Therefore, the creation of a national food composition database is urged globally [9]. Furthermore, nutritional data are needed to create processing parameters for the target items that are appropriate for low-income consumers [10].

Unfortunately, Ethiopia lacks its own national food database. The objective of this investigation was to assess the nutritional qualities and antinutritional factors of five wild fruits that are grown in Ethiopia, namely, *Rhus vulgaris*, *Rosa abyssinica*, *Rhus natalensis*, *Euclea racemosa*, and *Ficus sur*; the current study was carried out. These fruits are well-liked in Ethiopia and are a staple of the diet, especially in rural areas.

## 2. Methods and Materials

### 2.1. Plant Collection and Preparation

**Figure 1:**
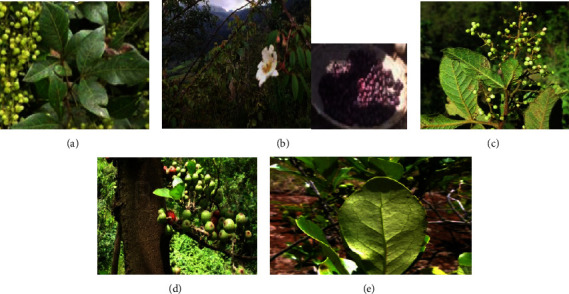
Wild edible fruit species for the current study: (a) *Rhus vulgaris*, (b) *Rosa abyssinica*, (c) *Rhus natalensis*, (d) *Ficus sur*, and (e) *Euclea racemosa.*

The Mekdela district, which is located at latitude 11° 25′ 25″N and longitude 39° 10′ 30″E, is where the fruit samples were taken from the wild forest. The district is located between 600 and 3700 meters above sea level. The district experiences 495 to 1168 mm of annual rainfall, and its average minimum and maximum temperatures range from 18 to 22°C, respectively. The specimens were identified by taxonomists from Mekdela Amba University. A voucher specimen was placed in the herbarium of the Department of Biology of Mekdela Amba University. The fruit samples were cleaned with distilled water to remove dirt, gently dried with a laboratory paper towel, cut into small pieces, and then dried (Figure 1). The dried materials were ground to a fine powder using an automatic motor blender, which was kept at 4°C in airtight glass containers until additional examination. All chemicals and reagents used in this investigation were of analytical grade and were brought from Addis Ababa. Wild edible fruits were dried for further analysis, and the fruit plant species *Rhus vulgaris*, *Rosa abyssinica*, *Rhus natalensis*, and *Euclea racemosa* fruits were dried by the solar drying while *Ficus sur* was dried by room drying (Table 1).

### 2.2. Proximate Composition and Antinutritional Factor Analysis

#### 2.2.1. Moisture Content

The moisture content of the fruit sample was determined according to the AOAC [11] standards. For each sample, 5.0 g of flour was first placed in a crucible and put in a drying oven at 105°C temperature, till a constant weight of the dried sample was attained. The moisture content (%M) was calculated using the following formula:

(1) $$\begin{array}{c} \text{moisture}\;\%=\frac{W3-W1}{W2-W1}\times 100, \end{array}$$

where *W*_1_ is the weight of the empty crucible, *W*_2_ is the weight of the sample and crucible, and *W*_3_ is the weight of the dry sample and the crucible.

#### 2.2.2. Crude Fat

For crude fat content determination, the Soxhlet extraction method was performed [12]. Two grams of moisture-free sample were introduced into a fat-free thimble and then placed in the extraction chamber. Hexane was added to the extraction equipment as a solvent due to its low boiling temperature, ease of oil recovery, and excellent solubilizing ability. After extraction, the solvent flask with fat and the thimble with the fat-free sample were removed from the apparatus. The solvent was evaporated after extraction and cooled in a desiccator. The crude fat was then calculated using the following formula:

(2) $$\begin{array}{c} \text{fat}\;\%=\frac{\text{weight}\;\text{of}\;\text{the flask}\;\text{with}\;\text{crude}\;\text{fat}-\text{weight}\;\text{of}\;\text{the flask}}{\text{weight}\;\text{of}\;\text{the sample}}\times 100. \end{array}$$

#### 2.2.3. Determination of Crude Protein

The crude protein content of the powdered fruit sample was determined by the micro-Kjeldahl method [13]. About, 5 g of fruit flour was first placed in a digestion tube along with 15 ml H_2_SO_4_ (98% pure) and 3 digestion tablets as a catalyst. The digestion was continued for 3-4 hours until a clear green color was obtained. After which, a 100 ml conical flask containing 20 ml of 40% boric acid and 3 drops of Tashiro's indicator was placed under the distillation apparatus with its outlet tubes implanted into the conical flask. The digest was washed away with distilled water by the addition of 3 drops of phenolphthalein and 20 ml of 40% (w/v) NaOH solution, and distillation was continued till about 50 ml of distillate was trapped into the boric acid plus indicator solution changed from red to light grey, displaying that all the ammonia liberated had been trapped. A receiving flask was titrated with 0.1 mM HCl to a brown color. Then, after titration, the % of nitrogen was determined as follows:

(3) $$\begin{array}{c} \text{nitrogen}\;\%=\frac{\left(V2-V1\right)\text{MHCl}\times N\times 1.4}{\text{weight}\;\text{of}\;\text{the sample}}\times 100,\\ \%\;\text{crude}\;\text{protein}=\%\;\text{nitrogen}\times 6.25, \end{array}$$

where *V*_2_ is the volume (ml) of HCl required to titrate the sample, *V*_1_ is the volume (ml) of acid required to titrate the blank, *M *is the molarity of acid, *N* is the normality of HCl, 6.25 is the protein nitrogen conversion factor, and 1.4 is the atomic mass of nitrogen.

#### 2.2.4. Crude Fiber

The crude fiber content was determined using AOAC methods [14]. First, 2 grams of moisture and fat-free fruit flour sample was digested with H_2_SO_4_ (1.25%) for 30 minutes followed by washing with distilled water and hydrolyzed for additional 30 minutes with 1.25% NaOH. The sample was washed with hot water and acetone and dried at 105°C for 1 hour till a constant weight was achieved and cooled in a desiccator and weighed. The sample remainder was ashed in a muffle furnace held at 550°C for 30 minutes and then cooled in a desiccator and weighed again. The crude fiber content was calculated using the following formula:

(4) $$\begin{array}{c} \text{fiber}\;\%=\frac{W1-W2}{W}\times 100, \end{array}$$

where *W*_2_ is the weight of (crucible + sample) after drying, *W*_1_ is the weight of (crucible + sample) after ashing, and *W* is the weight of the sample.

#### 2.2.5. Determination of Ash

Ash was determined using the standard AOAC methodology [15]. Two grams of each sample were added to porcelain crucibles and weighed and burned in a furnace for 30 minutes at 550°C. After being ashed, the samples were removed and allowed to cool in a desiccator before being weighed and then the percentage of ash is calculated as follows:

(5) $$\begin{array}{c} \text{ash}\;\%=\frac{W3-W1}{W2-W1}\times 100, \end{array}$$

where *W*_1_ is the weight of the crucible, *W*_2_ is the weight of the sample and the crucible, and *W*_3_ is the weight of the ash and the crucible.

#### 2.2.6. Total Carbohydrate Content

Carbohydrate content was determined by subtracting % moisture, % crude protein, % crude fat, % ash, and % crude fiber from 100.

#### 2.2.7. Energy Value

Total energy values were expressed in kcal/100 g and calculated as (percent crude protein × 4) + (percent fat content × 9) + (percent carbohydrate × 4).

### 2.3. Determination of Antiutritional Factors

#### 2.3.1. Oxalate Content

The amount of oxalate in the plant was determined using the modified titration method [16]. The material (1 g) had been ground and 75 ml of 3 mol/L of H_2_SO_4_ were added to a conical flask, which was then stirred for an hour using a magnetic stirrer. After filtering, 25 ml of the filtrate was collected and heated between 80° and 90°C. The temperature of this filtrate was consistently maintained above 70°C. After reaching the endpoint, which was shown by a light pink color that persisted for 15 seconds, the hot aliquot was continuously titrated with 0.05 mol/L of KMnO_4_. Calculating the oxalate content involved, assume that 1 ml of 0.05 mol/L of KMnO_4_ is equal to 2.2 mg of oxalate.

#### 2.3.2. Phytate Content

Phytic acid was found in the manner mentioned [17]. After weighing 2 grams of the sample into a flask and adding 100 ml of 2% HCl, the mixture was left to stand for 3 hours before being filtered. An indicator solution containing 0.3% ammonium thiocyanate was added to 5 ml of a separate 250 ml conical flask that contained 25 ml of filtrate. 53.5 ml of distilled water was added to achieve the appropriate level of acidity. Subsequently, this was titrated with a standard iron III chloride solution for five minutes until a brownish-yellow color permeated the mixture (0.001 95 g of iron per ml). The formula for phytic acid was found to be

(6) $$\begin{array}{c} \text{phytic}\;\text{acid}\left(\%\right)=\text{titer}\;\text{value }0.001\;95\times 1.19\times 100. \end{array}$$

#### 2.3.3. Determination of Saponins

As indicated in [18], the quantity of saponins was calculated. Essentially, 5 g of the powdered plant sample was combined with 50 ml of 20% ethanol, shaken for 30 minutes, and then heated for 4 hours at 55°C in a water bath. 200 ml of 20% aqueous ethanol was used to extract the residue once more after filtering the mixture. The filtrates were combined and diluted to 40 ml in a 90°C water bath. The concentrate was moved to a new funnel and vigorously agitated after 20 ml of diethyl ether was added. While the top layer, the ether layer, was being removed, the bottom layer, the aqueous layer, was held in a beaker. With a new funnel, the retained layer was added once more. After the separated layer was separated into an alternative funnel, 60 ml of n-butanol was added and stirred vigorously. The bottom layer was discarded, and the top layer known as butanol extract was kept. The butanol layer was washed twice with 10 ml of 5% aqueous sodium chloride. The remaining solution was collected, brought to a boil in a water bath to evaporate, and then dried in a drying oven at 40°C to a consistent weight. The following formula was used to determine the saponin content:

(7) $$\begin{array}{c} \%\;\text{saponin}\;\text{content}=\frac{\text{weight}\;\text{of}\;\text{the residue}\times 100}{\text{weight}\;\text{of the original}\;\text{sample}}. \end{array}$$

### 2.4. Determination of Mineral Contents

A sample of two grams of dry fruit flour was burned in a muffle furnace for three hours at 550°c after being charred on a hotplate until the smoking stopped. Weighed and dissolved in 3 ml of concentrated nitric acid, the resultant white ash was then diluted up to 25 ml with deionized water. Using atomic absorption spectroscopy grade standards, standard stock solutions of Ca, K, Zn, and Fe were prepared. The minerals Ca, Zn, and Fe were determined using an acetylene flame at wavelengths of 422.7, 213.9, and 248.3 nm, respectively, using an atomic absorption spectroscopy method [19]. For every mineral, a different set of electrode lights was utilized. The equipment was run for standard solutions of each mineral before and during determination to check that it was working properly. Using the same reagents and methodology as the samples and standards, blank solutions were made in order to evaluate any potential contamination. Atomic emission spectrometry operating at wavelengths of 766.5 nm was used to determine the potassium concentration.

(8) $$\begin{array}{c} \text{metal}\;\text{content}\left(\frac{\text{mg}}{100\;g}\right)=\frac{\left(C2-C1\right)\times V}{\left(10\times W\right)}, \end{array}$$

where *C*_2_ is the concentration of the sample in ppm (mg/L), *C*_1_ is the concentration of the blank in ppm (mg/L), *V* is the volume (ml) of the extract, and *W* is the weight (g) of the samples.

### 2.5. Data Analysis

The results of the nutritional composition and nutritional factor analyses of the ants were examined using one-way analysis of variance (ANOVA) techniques, presented as the mean ± SE of three measurements, and were determined statistically significant using SPSS version 20.

## 3. Results and Discussion

### 3.1. Proximate Composition

Table 2 shows the moisture content, ash, fat, protein, fiber, carbohydrate, and energy value of five wild edible plants. A considerable number of these proximate compositions were present in some edible wild plants. Among the studied edible plants, *Euclea racemosa* fruits contained high moisture content (16.24 ± 0.003 g/100) and ash content (12.8 ± 0.37 g/100) while it was observed to be low in terms of carbohydrate and energy composition. This result is comparatively higher than that of an earlier scenario in Ethiopia [7]. The higher moisture content indicates shorter storability if left unprocessed within a reasonable amount of time. It may be economically feasible to develop food products using the relatively high dry matter content of fruits, but it may not require a significant amount of raw material to produce a quantifiable amount of total solids [20].

The average carbohydrate content ranged from 65.54 ± 0.02 g/100 g in *Euclea racemosa* to 83.3 ± 0.28 g/100 g in *Rhus vulgaris*, which corresponds to approximately the recommended daily allowance (RDA) of carbohydrates (130–210 g/day) for all age groups [21]. This indicates that fruits have a high potential to provide sufficient amounts of the body's main energy source. The highest and lowest protein contents of the fruits were recorded, with *Rhus natalensis* (0.98 ± 0.003 g/100 g) and *Euclea racemosa* (3.22 ± 0.01 g/100 g), respectively. The protein content of *Euclea racemosa* in this investigation is significantly higher than the 1% reported by [2], which was carried out in Northeast India. Depending on the age group, these fruits can contribute between 16% and 133% of the required protein due to their inherent protein values [22].

The ash content, which ranged from 2.41 ± 0.01 g/100 g in *Rhus vulgaris* to 12.8 ± 0.37 g/100 g in *Euclea racemosa*, indicates its mineral strength and, if consumed, can greatly decrease some micronutrient deficiencies that have a negative impact on human health. This finding is similar to the fruits of *Ziziphus nummularia* and *Ziziphus mauritiana* that are carried out in India [23]. Compared to other studies reported by [24] in Ghana, the fat content ranged from 0.12 ± 0.003 g/100 g for *Ficus sur* to 0.9 ± 0.00 g/100 g for *Rosa abyssinica*, which was very low. The low fat content could indicate that fruits are crucial in reducing cardiovascular disease.

The crude fiber content ranged from 1.53 ± 0.03 g/100 g in *Euclea racemosa* to 9.54 ± 0.003 g/100 g in the *Rhus natalensis* fruits. This corresponds to approximately 5–22% of the recommended daily allowance (RDA) of fiber for humans, which is 19–38 g [25]. This quantity of fiber may have a significant impact on the way humans digest food and manage constipation. *Rhus natalensis* had an energy content of 274.5 ± 0.07 kcal/100 g, while *Rhus vulgaris* had 344.5 ± 2.21 kcal/100 g. This indicates that when consumed, fruits could provide about 11 to 19% of daily energy needs (2071 to 3152 kcal) [26].

### 3.2. Mineral Composition

**Figure 2:**
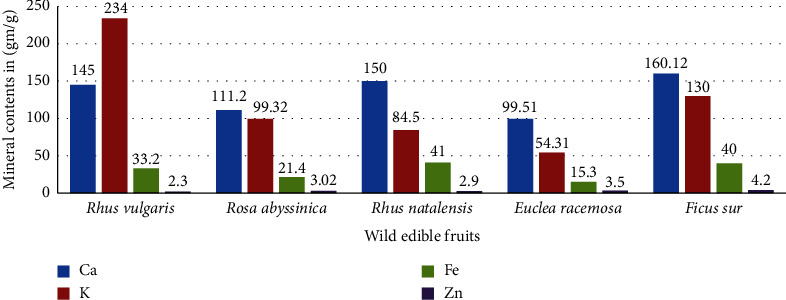
Mineral composition of edible wild fruits in DW (mg/100 g).

The presence of trace minerals (Fe and Zn) and macrominerals (K and Ca) was investigated in terms of dry matter, as shown in Figure 2. In *Euclea racemosa*, the calcium content ranged from 99.51 mg/100 g to 160.12 mg/100 g in *the sur*. Calcium must be included in human diets because it is essential for the development of strong bones and teeth, the control of muscle contractions, and the transmission of nerve impulses throughout the body [27]. *Rhus vulgaris* had the highest potassium concentration of 234 mg/100 g, while *Euclea racemosa* had the lowest concentration of 54.34 mg/100 g. As a result, there was a statistically significant variation in potassium concentration between fruits that was statistically significant (*p* > 0.05). Potassium is an essential mineral that helps the body to maintain its balance of osmotic and bodily fluids and to regulate nerve signals and muscle contractions [28].

Furthermore, an appreciable amount of zinc was found from 2.3 mg/100 g of *Rhus vulgaris* to 4.2 mg/100 g of *Ficus sur. Rhus natalensis* had a higher iron concentration (41 mg/100 g). Zinc is significantly associated with protein synthesis, catalytic activity of several enzymes, and rapid growth and development during childhood, adolescence, and wound healing, while iron is extremely important for oxygen transport capacity and for controlling anemia and its associated consequences [29].

### 3.3. Antinutrient Composition

**Figure 3:**
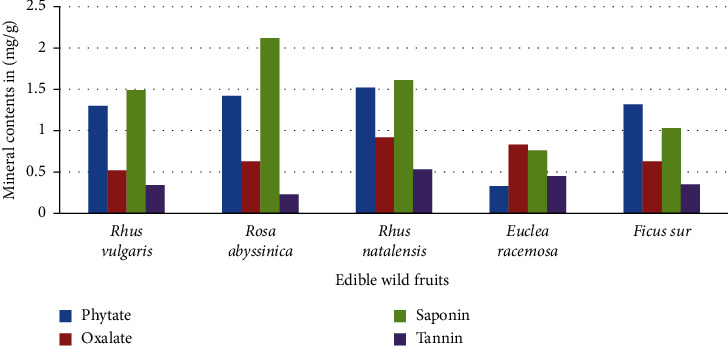
Antinutrient content (mg/g) of five edible wild fruits.

*Rhus natalensis* had a higher phytate content (1.52 mg/100 g), although *Euclea racemosa* had the lowest content (0.33 mg/100 g) as shown in Figure 3. Phytate decreases the digestibility of amino acids and forms complexes with phosphorus, calcium, magnesium, iron, and zinc to obstruct their absorption. As a result, the body can easily become unavailable to these minerals [30]. The oxalate values ranged from 0.52 mg/100 g in *Rhus vulgaris* to 0.92 mg/100 g in *Rhus natalensis.* Renal calcium absorption is known to be inhibited by oxalate, particularly at concentrations of approximately 4-5 g/100 g [31]. However, the values found in this investigation are significantly lower than the values that are considered hazardous. This implies that eating fruits may not cause any problems with the absorption of minerals by the body [32]. The saponin content ranged between 0.76 mg/100 g in *Euclea racemosa* and 2.12 mg/100 g in *Rosa abyssinica*. When saponins are present in large amounts, they can impart their distinctive bitter taste to food and cause them to foam in aqueous solution [33]. Although saponins rupture red blood cells and induce nausea and vomiting, they help people with hypocholesterolemia [34].

The tannin content ranged from 0.23 mg/100 g in *Rhus natalensis* to 0.53 mg/100 g in *Rhus natalensis*, which is less than that reported by [35] for similar forest fruits. Due to its astringent properties, tannin has been demonstrated to decrease food palatability, impede the activity of certain enzymes, and combine with proteins to decrease their solubility and digestibility. However, these fruits may not present any major health risks due to their low tannin content [36].

### 3.4. Comparison of Determined and Reported Nutritional Values of Wild Edible Fruits

Many national and international researchers have documented the nutritional makeup of various wild edible fruit species. The most frequently reported nutritional parameters are ash, moisture, crude protein, crude fat, crude fiber, crude carbs, and energy.  Table 3 compares the values obtained in this investigation with those reported in the literature. It is evident from the table that there were notable differences in the nutritional makeup of the same parameters reported by various researchers. The present investigation found that the moisture content of *Rosa abyssinica* fruit (13.24 ± 0.003 g/100 g) was comparable to that of *Ziziphus spina-christi* (13.10%), which was described by the authors in [37] as underutilized wild edible fruits in the East Wollega zone, Western Ethiopia. The crude fiber content in this study was significantly lower than the previous finding [38] which recorded 20.46% in *Mimusops kummel* fruit in the Amhara region of Ethiopia. Researchers may have used different methodologies for sample preparation and analysis, which could account for the disparity. The fat content of the current study was almost exactly the same as the values for naturalized wild edible fruit species in Ethiopia reported by Mokria et al. [39]. The fruit of *Rhus vulgaris* has the highest carbohydrate content (83.3 ± 0.28 g/100 g). Furthermore, this finding was also in agreement with a study conducted by Mahapatra et al. [40], who reported 12.43% in *Carissa spinarum* fruit. Genetic and environmental factors could be the cause of this variation. The energy value in *Rhus natalensis* fruit (274.5 ± 0.07 kcal/100 g) was closely related to Yimer et al. [41], who reported (275.0 ± 5.9 kcal/100 g) the total energy value in *Solanum nigrum* fruit.

## 4. Limitation of the Study

The toxicity, antioxidant activity, fatty acid composition, and phytochemical analyses for each of the five wild edible fruit species were not complete due to a lack of funding and a well-equipped laboratory for this work.

## 5. Conclusions

According to their recommended proximate and mineral contribution to daily nutrient requirements in humans, *Rhus vulgaris*, *Rosa abyssinica*, *Rhus natalensis*, *Euclea racemosa*, and *Ficus sur* are nutritionally sound. Fruits may not present significant health risks due to their relatively low levels of antinutrients. Therefore, it is clear from the study that the nutritional and antinutritional factors of wild edible fruits, despite their abundance and lack of public acceptance and use in the Ethiopian community, could be used as a weapon against hunger and certain nutrient deficiencies. To guarantee widespread consumption and antioxidant properties, it would be highly helpful to process seasonal and perishable fruits into unique and complete food products, such as drinks, baked goods, or cookies.

## Figures and Tables

**Table 1:** Duration of time for wild edible fruits drying.

Fruit species	Method of drying	Plant part	Period of drying
*Rhus vulgaris*	Solar drying	Fruit	3 days
*Rhus natalensis*	Solar drying	Fruit	2 days
*Rosa abyssinica*	Solar drying	Fruit	3 days
*Euclea racemosa*	Solar drying	Fruit	5 days
*Ficus sur*	Room drying	Fruit	6 days

**Table 2:** Proximate composition of five wild edible fruits.

Parameters	Proximate analysis of five wild edible fruits (g/100 g dry weight)				
	*Rhus vulgaris*	*Rosa abyssinica*	*Rhus natalensis*	*Euclea racemosa*	*Ficus sur*
Moisture	10.8 ± 0.03^b^	13.24 ± 0.003^c^	14.21 ± 0.01^d^	16.24 ± 0.003^e^	10.12 ± 0.003^a^
Total ash	2.41 ± 0.01^a^	5.12 ± 0.003^b^	8.22 ± 0.01^c^	12.8 ± 0.37^d^	8.20 ± 0.00^c^
Crude fat	0.45 ± 0.03^d^	0.9 ± 0.00^d^	0.48 ± 0.02^b^	0.71 ± 0.01^c^	0.12 ± 0.003^a^
Crude fiber	1.58 ± 0.02^a^	1.73 ± 0.03^b^	9.54 ± 0.003^d^	1.53 ± 0.03^a^	2.02 ± 0.006^c^
Crude protein	2.23 ± 0.03^d^	1.2 ± 0.00^b^	0.98 ± 0.003^a^	3.22 ± 0.01^e^	2.02 ± 0.003^c^
Carbohydrates	83.3 ± 0.28^d^	77.8 ± 0.03^c^	66.54 ± 0.02^b^	65.54 ± 0.35^a^	77.52 ± 0.01^c^
Energy	344.5 ± 2.21^d^	324.1 ± 0.14^c^	274.5 ± 0.07^a^	281.35 ± 1.4^b^	319.27 ± 0.04^c^

*Note.* Values are the averages of three separate composite sample analyses (based on DW) that were performed independently. The superscripts that vary along the column indicate significant differences at *p* < 0.05.

**Table 3:** Comparison of observed nutritional compositions of wild edible fruit samples with reported values.

Fruits species	Moisture	Ash	Protein	Fiber	Fat	Carbohydrate	Energy value	References
*Ziziphus spina*-*christi*	13.10 ± 0.02^a^	9.23 ± 0.36^a^	5.31 ± 0.01^a^	0.71 ± 0.02 d	1.65 ± 0.29^c^	70.41 ± 0.33^d^	316.05 ± 1.43^c^	[37]
*Solanum nigrum*	6.0 ± 0.6^b^	14.0 ± 0.4^b^	21.7 ± 0.9^a^	22.3 ± 0.4^a^	4.0 ± 0.6^b^	38.1 ± 1.2^e^	275.0 ± 5.9^c^	[41]
*Carissa spinarum*	73.2 ± 1.37	ND	3.64 ± 1.04	ND	ND	12.43 ± 0.39	ND	[40]
*Mimusops kummel*	13.10	2.60	2.19	20.46	1.62	80.49	ND	[38]
*Cordia africana*	11.15 ± 0.34	15.88 ± 2.58	10.30 ± 2.60	3.86 ± 0.34	0.69 ± 0.16	60.26 ± 3.92	277.97 ± 10.88	[39]
*Rhus vulgaris*	10.8 ± 0.03^b^	2.41 ± 0.01^a^	2.23 ± 0.03^d^	1.58 ± 0.02^a^	0.45 ± 0.03^d^	83.3 ± 0.28^d^	344.5 ± 2.21^d^	Current study
*Rosa abyssinica*	13.24 ± 0.003^c^	5.12 ± 0.003^b^	1.2 ± 0.00^b^	1.73 ± 0.03^b^	0.9 ± 0.00^d^	77.8 ± 0.03^c^	324.1 ± 0.14^c^	Current study
*Rhus natalensis*	14.21 ± 0.01^d^	8.22 ± 0.01^c^	0.98 ± 0.003^a^	9.54 ± 0.003^d^	0.48 ± 0.02^b^	66.54 ± 0.02^b^	274.5 ± 0.07^a^	Current study
*Ficus sur*	10.12 ± 0.003^a^	10.12 ± 0.003^a^	2.02 ± 0.003^c^	2.02 ± 0.006^c^	0.12 ± 0.003^a^	77.52 ± 0.01^c^	319.27 ± 0.04^c^	Current study

ND: not determined.

## Data Availability

The data used to support this study are included in the article.
